# Quality Evaluation of *Corydalis yanhusuo* by High-Performance Liquid Chromatography Fingerprinting Coupled with Multicomponent Quantitative Analysis

**DOI:** 10.1038/s41598-020-61951-x

**Published:** 2020-03-19

**Authors:** Yin Lu, Qin Ma, Changchun Fu, Chuan Chen, Deyong Zhang

**Affiliations:** 1 College of Biology and Environmental Engineering, Zhejiang Shuren University, Hangzhou, 310015 China; 2 Hangzhou Botanical Garden, Hangzhou, 310007 China

**Keywords:** Secondary metabolism, Conservation biology

## Abstract

Corydalis Rhizoma is the tuber of *Corydalis yanhusuo* W. T. Wang, which has been long used in traditional Chinese medicine. Herein, the quality of *C. yanhusuo* samples collected from 23 regions of three provinces in China is evaluated through high-performance liquid chromatography fingerprinting coupled with similarity, hierarchical clustering, and principal component analyses. Sample similarities are evaluated according to the State Food and Drug Administration requirements by selection of 18 characteristic chromatographic fingerprint peaks and are found to vary between 0.455 and 0.999. Moreover, common patterns of a typical local variety of *C. yanhusuo* sourced in the Panan County are established. The obtained results show that the combination of quantitative analysis and chromatographic fingerprint analysis can be readily utilized for quality control purposes, offering a comprehensive strategy for quality evaluation of *C. yanhusuo* and related products.

## Introduction

The growing popularity of traditional Chinese medicines (TCMs) due to their high effectiveness and low toxicity has drawn the increased attention of the scientific community^1^. However, because of the complex chemical composition of TCMs, the quantity and quality of related safety and efficacy data are insufficient to meet the criteria required for global usage^2^, which highlights the need for methods of effective TCM composition elucidation and quality evaluation. Chromatographic fingerprinting, a comprehensive and quantifiable identification method, relies on the processing of holistic chemical profiles of botanical extracts by analytical and chemical techniques^3^. Chromatographic fingerprinting coupled with quantitative analysis, exhibiting the benefits of entirety and fuzziness and used to evaluate herbal product quality consistency and stability, can be used to characterize both marker compounds and unknown components of complex samples and has been widely employed for TCM authenticity identification, origin differentiation, and quality evaluation^4–6^. At present, this method has been adopted by the World Health Organization and other authorities as a strategy for assessing the quality of herbal medicines^7^ and is also a part of industry’s widely accepted model of quality level evaluation^8^. Among the chromatographic fingerprinting methods used for TCM authentication and qualitative evaluation over the past decade, high-performance liquid chromatography (HPLC) fingerprinting is most widely employed to discriminate the origins of medicinal herbs and conduct TCM quality control owing to its convenience and efficiency^9,10^.

The dried tuber of *Corydalis yanhusuo* W. T. Wang (Corydalis Rhizoma, or Yuanhu in Chinese) is a well-known TCM that has been long used to promote blood circulation, reinforce vital energy, inhibit cancer cell proliferation, and alleviate pain^11^, and can also be applied to treat cardiovascular diseases^12^. Among the hundreds of isolated *C. yanhusuo* metabolites such as fatty acids, organic acids, amino acids, sugars, steroids, anthraquinones, and volatile compounds^13–16^, alkaloids are known to be the major pharmacologically active compounds for the treatment of blood vessel diseases, tumors, and various pains^17,18^. The main (~60) specifically active alkaloids, including tetrahydropalmatine (THP), corydaline, protopine, columbamine, berberine, dehydrocorydaline (DHC), tetrahydrocolumbamine, and palmatine were identified by *in vivo* screening tests on animal models (rats) or human cancer cells^19–21^. To date, up to now, as in the cases of most herbal medicines, current studies on *C. yanhusuo* focus on the isolation and therapeutic function determination of the alkaloids (e.g. tetrahydropalmatine and its tertiary-amine alkaloid derivatives) of interest^22^, whereas the combination of HPLC fingerprinting with multi-ingredient quantitation for the quality control of *C. yanhusuo* are less well reported. Therefore, complete profiling of this TCM originating from different geographic regions through chemical fingerprinting and multicomponent quantitative analysis is of great interest and importance. Moreover, the chemical components of *C. yanhusuo* are dependent on with and without pre-preparation process. In order to increase the comparability, the fingerprints of processed (e.g. boiled and sulfur)^23^, and unprocessed (raw) herb should be established respectively.

Herein, an effective HPLC fingerprinting method coupled with hierarchical clustering analysis (HCA) and principal component analysis (PCA) is established for the identification and quality evaluation of processed and raw *C. yanhusuo* materials from different origins. This technique allows one to comprehensively probe the (quantitative) chemical composition and similarities of *C. yanhusuo* from different geographic locations and processes, which can therefore be of great use for the further study and development of this TCM.

## Results and Discussion

### Extraction condition optimization

The tubers of *C. yanhusuo* were selected, which were irregular and oblate in appearance and 1~2 cm in diameter. The best tubers were big, plump, solid, yellow and bright in inner color. To maximize extraction efficiency and the amount of fingerprint information, we optimized extraction conditions using a univariate approach, which included the optimization of extraction solvent (methanol, 70 vol% aqueous ethanol, 70 vol% aqueous ethanol + 0.2 vol% acetic acid) and number of extractions (1, 2, or 3). The number and area of chromatographic peaks were maximal for 70 vol% ethanol, which was therefore selected as the extraction solvent. The influence of the number of ultrasonication-assisted extractions on extraction efficiency was investigated by extracting powdered samples with 70 vol% ethanol for 1, 2, or 3 times (30 min at a time). Extraction efficiency is increased with the number of extractions, but the peak areas of target compounds did not significantly increase after two times. Thus, the optimal extraction condition was established as 2 × 30 min ultrasonication-assisted extraction with 70 vol% aqueous ethanol.

### HPLC condition optimization

Detection wavelength, column temperature, flow velocity, mobile phase, and gradient elution procedure were optimized to obtain as much chemical information as possible and achieve the best separation of adjacent peaks in the fingerprint chromatograms of *C. yanhusuo* within a short time. Based on the maximum absorption and full-scan experiment data of marker components in the UV spectra of three-dimensional chromatograms obtained by photo-diode array detection, detection wavelengths in the range of 210~350 nm (especially 210, 254 and 280 nm) were tested to maximize the number and resolution of all marker compound peaks. As a result, the peak height varies greatly with the wavelength change. Considering the appropriate peak height of the reference material and the chromatogram of the samples, 280 nm was chosen to record characteristic chromatographic patterns and improve the corresponding baseline. For resolution enhancement, the column temperature (30 or 40 °C) and mobile phase flow velocity (1 or 0.8 mL/min) were considered, and the best peak separation and shape were obtained at 40 °C and 1 mL/min. The investigation of the effect of mobile phase composition (methanol-water and acetonitrile-water with different modifiers such as acetic acid and triethylamine) on chromatographic separation showed that although the peak number was maximized in the case of acetonitrile-water, this system could not achieve satisfactory separation. As the addition of acidic modifiers can increase alkaloid peak separation and minimize peak tailing by restraining ionization^24^, acetic acid was added to the acetonitrile-water system to further improve peak shape. As a result, the best peak resolution/separation, sensitivity, and selectivity were obtained in the presence of 0.06 vol% triethylamine and 0.06 vol% acetic acid at pH 4.0. In the process of gradient optimization, which involved the testing of different gradient time, procedures, and initial mobile phase compositions, the best separation was attained within 90 min using the optimized procedure.

### Validation of the quantitative analysis method

The developed method was validated in terms of precision, stability, and reproducibility. Moreover, a well-linear relationship between the peak area and concentration of each reference compound (*R*^2^ > 0.9998) was observed for all analytes within the test range. For precision evaluation, a sample (S6) of medicinal material of *C. yanhusuo* prepared as described above was subjected to five-fold HPLC analysis on the same day. Stability was analyzed in 0, 2, 4, 6, 12, and 24 h within two days, and repeatability was examined by injection of five different samples prepared using the same procedure. Variations were expressed as relative standard deviations (RSDs). Precision based on the relative retention time of common peaks was in the range of 0.07–0.50%, and that based on bioactive component peak areas was in the range of 0.67–2.27%. The stability (RSD, *n* = 5) of measurements over two days ranged from 0.15 to 0.61% of the relative retention time and from 0.72 to 3.27% of the peak area. In the case of reproducibility, the RSDs of relative retention time and peak area varied from 0.04 to 0.46% and from 1.00 to 3.68%, respectively. Thus, the results showed that the developed method was sufficiently precise and accurate (RSD < 3%) for quantitative profiling of *C. yanhusuo* from different regions, meeting the technical requirements of fingerprinting.

### HPLC fingerprint establishment and similarity analysis (SA)

**Figure 1:**
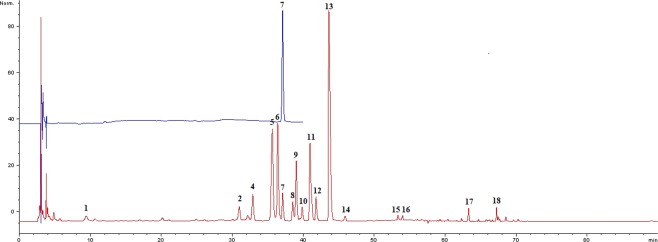
Position of the THP peak in the fingerprint.

*Calibration of THP in HPLC fingerprint*. However, the study didn’t identify these characteristic chromatographic fingerprint peaks for *C. yanhusuo* samples except THP, which was used as the only identification control component specified in Pharmacopoeia of the People’s Republic of China^25^. As THP is one of the most important active constituents of *C. yanhusuo*^26^, its peak was chosen as a reference (retention time = 37 ± 1 min, Peak 7 in Fig. 1).

#### Analysis and comparison of HPLC fingerprints

**Figure 2:**
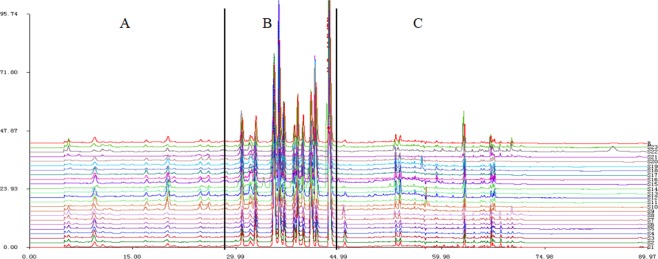
HPLC fingerprints of the 23 *C. yanhusuo* samples (S1–23, Table 1) and the reference fingerprint (R) obtained by the Similarity Evaluation System for Chromatographic Fingerprint of Traditional Chinese Medicine software (Version 2004A, Chinese Pharmacopoeia Committee, Beijing, China).

The chromatographic fingerprints of 23 *C. yanhusuo* samples originating from different locations and processed by different methods are presented in Fig. 2. In general, characteristic peak selection was based on the criterion that peaks found in each chromatogram of variable-location samples should be well separated under the given chromatographic conditions and have relatively large areas in different profiles^27^. The reference fingerprint (marked as R in Fig. 2) was established as the median of 23 chromatograms to identify and evaluate the quality of *C. yanhusuo*, and 18 peaks were extracted as characteristic common peaks. To facilitate identification and analysis, the whole chromatogram was divided into three areas, namely area A (retention time 0–29.0 min), area B (retention time 29.1–45.0 min), and area C (retention time 45.1–90 min). Among the fingerprints of these areas, that of area B was subject to least change and exhibited obvious characteristics, which allowed area B to be used as a characteristic area of *C. yanhusuo*. Conversely, the fingerprints of areas A and C were broadly variable, featuring chromatographic peaks and peak areas dependent on sample origin and processing method, which could be used as the basis for the identification of this medicinal material.

#### Effects of processing on HPLC fingerprints

**Figure 3:**
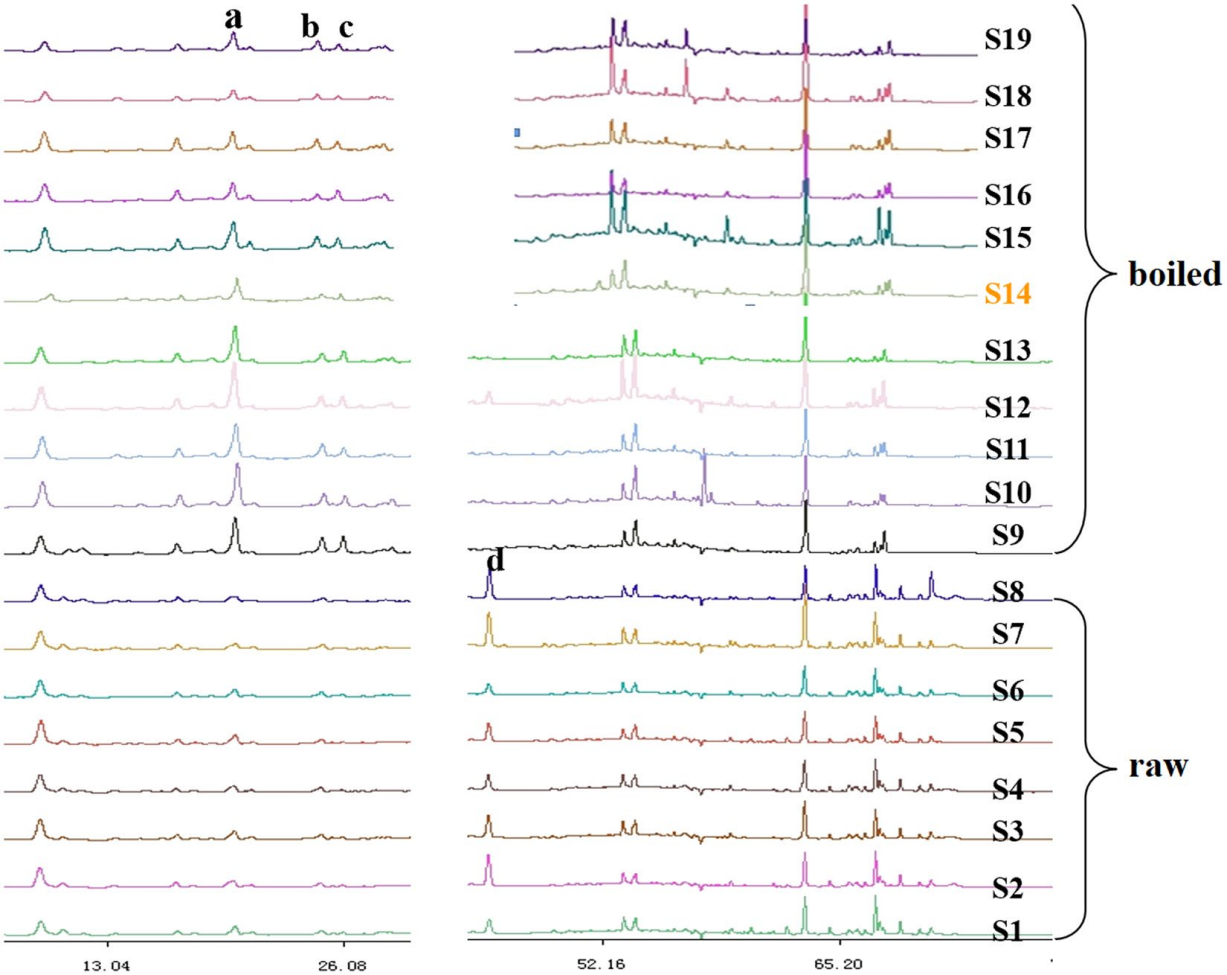
Differences of areas A and C in the fingerprints of *C. yanhusuo* (raw & boiled).

Samples 1–8 and 22–23 were unprocessed (crude drugs), 9–20 were boiled (cooked drugs), and sample 21 was sulfur-fumigated. For better comparison, we used samples originating from Zhejiang, excluding those produced outside of this province (samples 1–20). It seemed the fingerprints of crude drugs and cooked medicines were obviously different in areas A and C (see Supplementary Fig. S1). As shown in Fig. 3 for area A, the areas of cooked material peaks at ~20 min (peak a), 25 min (peak b), and 26 min (peak c) obviously exceeded those of raw materials, indicating that the corresponding component contents were also higher. For area C, a small peak at ~43 min (peak d) was observed for raw materials, whereas no peaks were found in the case of cooked materials (except for sample 12). The spectra of cooked materials were markedly different from those of raw materials in the retention time range of 55–70 min, e.g., at times above 65 min, almost no peaks were observed for the former materials, whereas some small peaks were detected for the latter.

**Figure 4:**
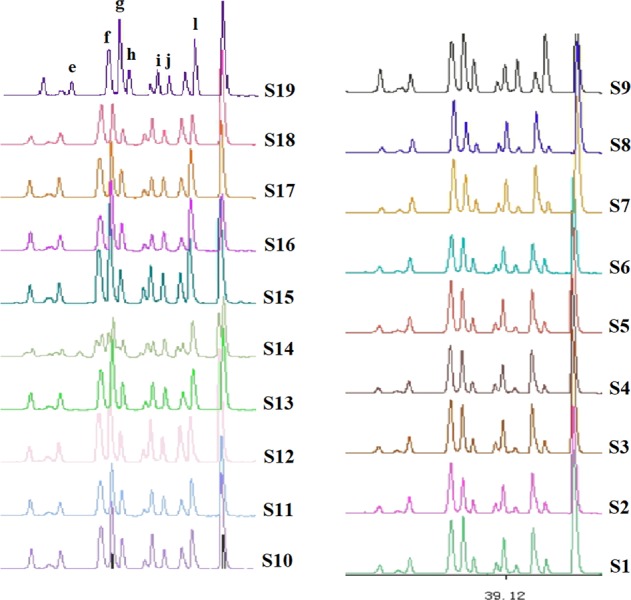
Differences of area B in fingerprints of *C. yanhusuo* (raw & boiled).

In addition to the obvious differences between areas A and C, the fingerprint patterns of area B also changed in shape, mainly as follows. Upon going from raw to boiled materials, peak e (~32.01 min) changed from single to double, and the shape of peaks f–h (35–37 min) changed from a decreasing-height ladder to a middle one. Peak i (39.0 min) was similar to peak j (39.8 min) for boiled materials, but the relative heights of these peaks were significantly different for raw materials. The intensity of peak k (40.9 min) exceeded that of peak l (41.7 min) for raw materials, whereas the reverse was true for boiled materials (Fig. 4).

Sample 21, *C. yanhusuo* produced in Dongyang, Zhejiang Province, and processed by sulfur fumigation, was compared with the boiled material produced in the same location to facilitate comparison. Notably, the former sample was found to contain less constituents than the latter, e.g., peaks a (~20 min), m (~53 min), n (~54 min), and o (~63 min) were absent in the former case (see Supplementary Fig. S2). Therefore, it was speculated that sulfur fumigation may decrease the quality of *C. yanhusuo* medicinal materials.

#### Effects of sample origin on HPLC fingerprints

**Figure 5:**
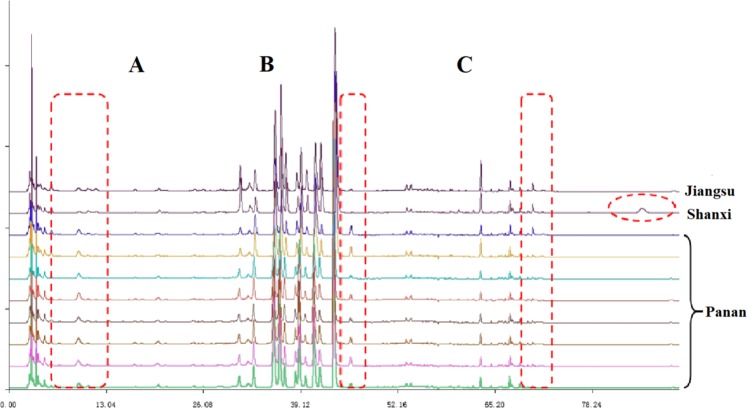
HPLC fingerprints of *C. yanhusuo* of different geographical origin.

The Panan County of Zhejiang Province is the main area of *C. yanhusuo* medicinal material production, with the corresponding output accounting for more than 30% of the national market and traditionally featuring excellent quality. Therefore, we systematically investigated the fingerprints of *C. yanhusuo* produced in Panan, Shanxi, and Jiangsu counties to provide guidelines for the selection of high-quality medicinal materials for the Chinese medicine industry and clinical use. For uniform comparison, unprocessed medicinal materials were used for fingerprint analysis (Fig. 5), which revealed that samples produced in Panan was different from those produced elsewhere. In the case of Shaanxi and Jiangsu samples, peaks in areas A and B were not obvious or absent, whereas a small peak was observed in each of these areas for Panan samples. In area C, no peaks were observed for Panan samples, whereas a minor peak was observed for samples of other origins.

#### SA of fingerprints of different origins

As the State Food and Drug Administration suggest that all herbal chromatograms should be evaluated in terms of similarity by calculation of the correlation coefficient and/or angle cosine values of the original data^28^, SA was herein performed to evaluate the similarities and differences between *C. yanhusuo* samples. As showing in Fig. 3, the chromatographic profiles of the tested samples were generally consistent, although the intensities of some peaks and peak number were subject to some variation. The similarities between the generated reference fingerprint and individual sample fingerprints were calculated as 0.455–0.999 using the similarity evaluation system (can be found as Supplementary Table S1). The similarity values of 21 samples exceeded 0.9, i.e., similar components were present in these samples regardless of geographic location. However, low similarity values of <0.9 observed for samples 14, 16, 20, and 21 suggested that the compositions of these samples might be different from those of samples with high similarity values. Thus, crude herbs and prepared slices were concluded to be significantly different because of the production process and the variable contents of main bioactive constituents. According to Pharmacopoeia of the People’s Republic of China, THP was used as the identification control component. In terms of THP content, S5 was the highest in fresh samples and S17 was the highest in boiled samples. The chemical composition of *C. yanhusuo* is complex, and its efficacy is not the simple function of one or several effective substances, nor is the higher the content of some components, the better its quality, but depends on the composition and proportion of the whole chemical composition of *C. yanhusuo*. Therefore, we also need to combine pharmacodynamic research and the correlation analysis of the material basis of secondary metabolites, we can tell which sample was be ‘reference’ of good quality.

### Qualitative determination of the HPLC fingerprint of *C. yanhusuo* by chemometrics

#### HCA

It is a technique used to sort samples into groups^29^, has been widely applied to fingerprint analysis, as it permits simple nonparametric data interpretation^30^ and provides a visual representation of complex data. Herein, a method known as average linkage between groups was applied, and Pearson correlation was selected as a measurement. The applied HCA method classified different herbs by measuring the areas of generic characteristic peaks processed by the similarity evaluation system^31^.

**Figure 6:**
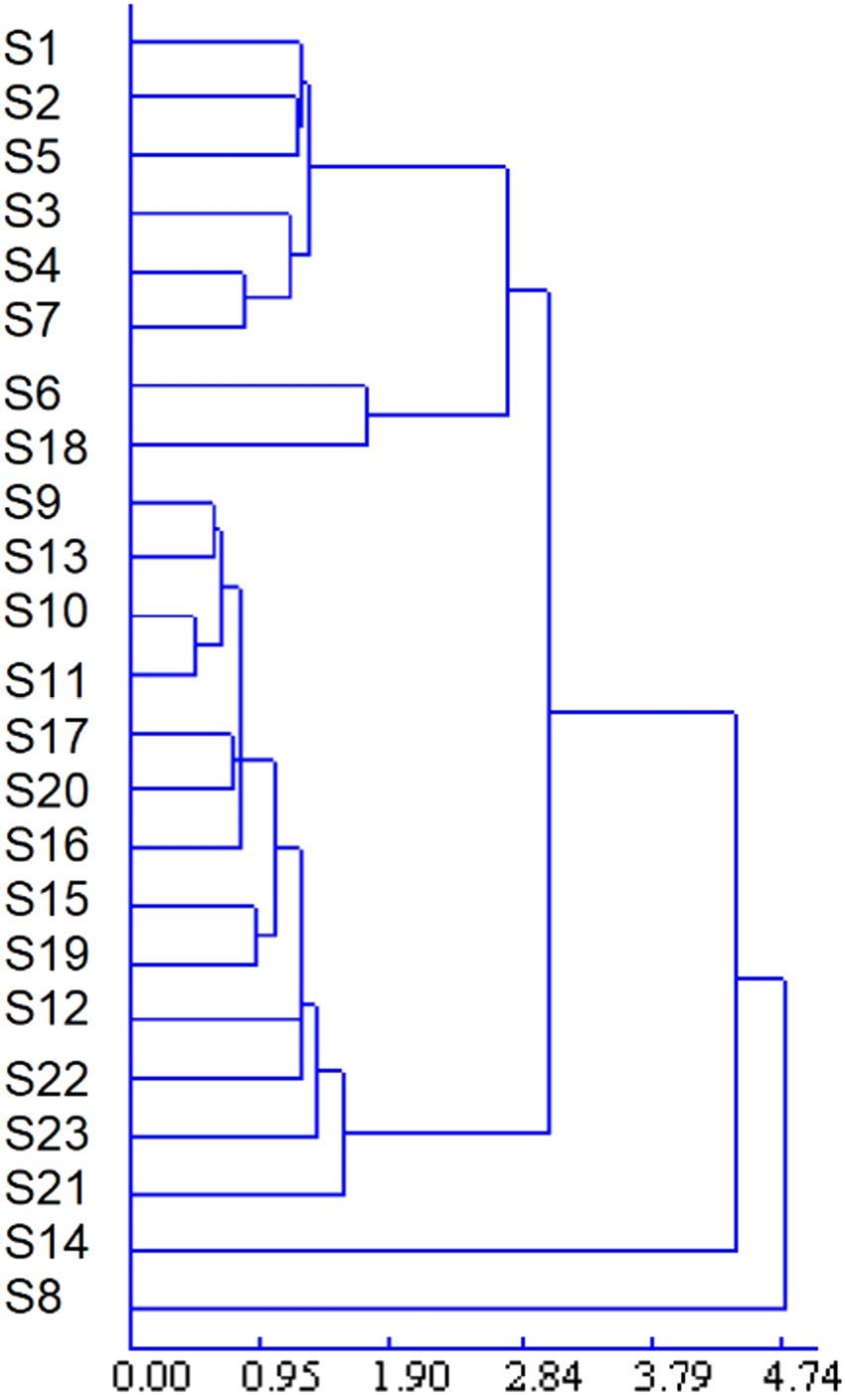
HCA dendrogram for the 23 *C. yanhusuo* samples.

To assess the resemblance and differences between different *C. yanhusuo* samples as a whole, hierarchical agglomerative clustering analysis (based on Euclidean distance measurement and shortest-distance clustering, DPS V8.01) of the 23 *C. yanhusuo* samples was performed based on the relative areas of characteristic peaks. The obtained results (Fig. 6) clearly showed that most Panan samples (except S14 and S8) were clustered together. Shanxi (S22) and Jiangsu (S23) samples were different from others, and S21 (sulfur-fumigated) was classified into a separate group. S14 was considered to have undergone composition changes during boiling. The similarity between S8 and other samples exceeded 0.9, but these two sample groups were still treated as part by cluster analysis, and the exact source and quality of S8 were therefore concluded to deserve further analysis. HCA results were validated against each other and provided further references for the quality evaluation of *C. yanhusuo*.

#### PCA

**Figure 7:**
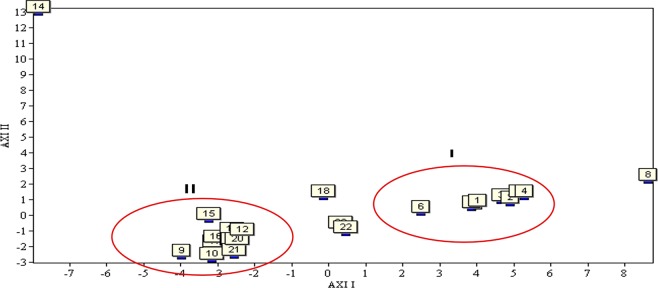
PCA results for the 23 *C. yanhusuo* samples.

Which is an unsupervised multivariate data analysis approach, is applicable when a function of many attributes is believed to be involved in different samples^32^. Herein, PCA was employed to analyze the relationships between the 23 *C. yanhusuo* samples of different origin, projecting them to low-dimensional space to observe subtle differences. The resulting score plot is presented in Fig. 7.

### Establishment of a standard HPLC fingerprint of *C. yanhusuo* from Zhejiang Province

#### Typical fingerprint selection

By studying the fingerprints of 23 batches of *C. yanhusuo* and combining their characteristics with other physicochemical identification data, we selected 16 batches (S1–7, S9–13, S15, S17–19) of typical *C. yanhusuo* samples produced in Panan and constructed their standard fingerprints.

#### Relative retention time and relative areas of common peaks

**Figure 8:**
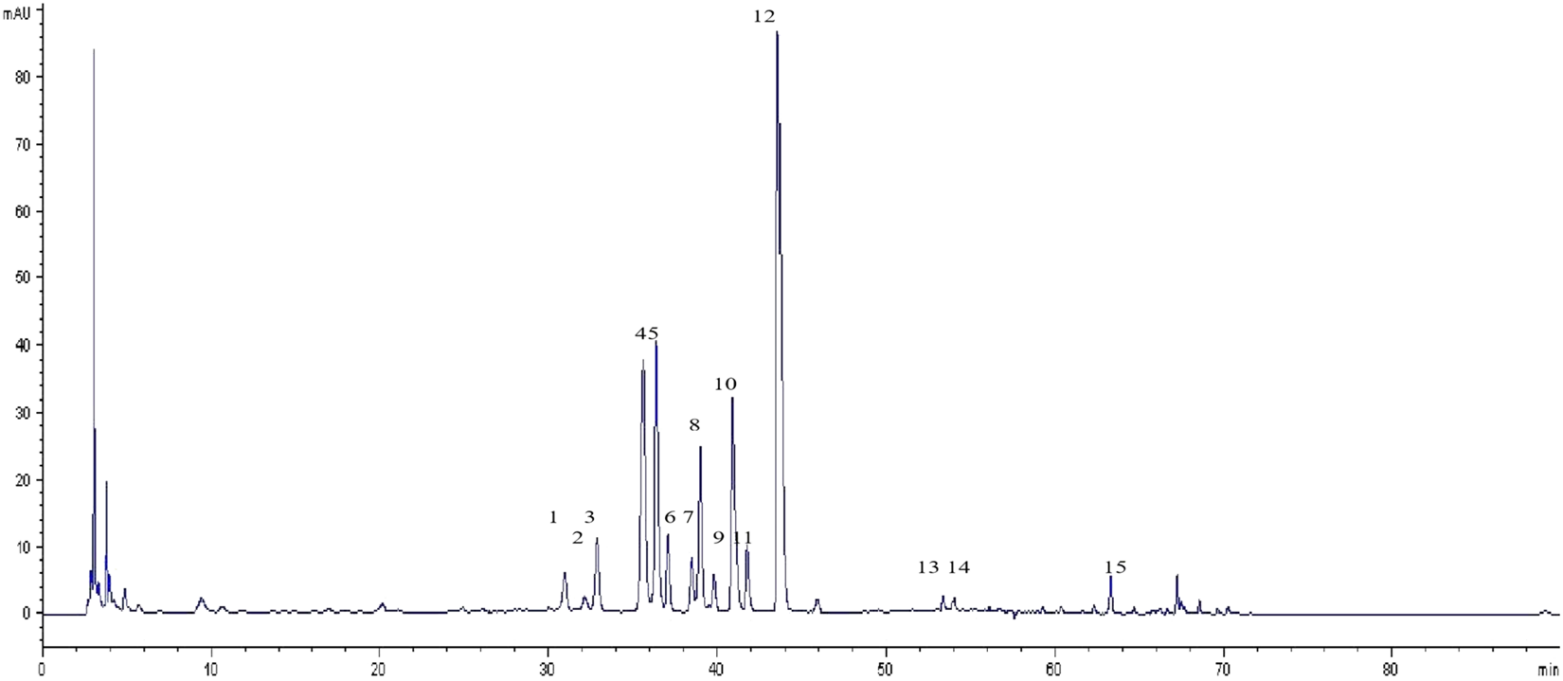
Characteristic peaks of *C. yanhusuo* fingerprints.

After comparison and analysis, 15 common peaks of *C. yanhusuo* medicinal material were identified (Fig. 8), with peaks 5, 6, 9, 11, and 13 accounting for more than 5% of the total peak area. The total peak area of each batch of chromatographic fingerprints exceeded 90%, in compliance with fingerprinting requirements. The relative retention time of common peaks can be found as Supplementary Table S2.

#### Generation of common patterns

Peaks present in the chromatograms of all samples with reasonable height and good resolutions were denoted as common peaks (in the case of single peaks) or common patterns (in the case of a region containing several peaks). Analysis of a batch of chromatographic fingerprints allowed us to simulate control fingerprint data or control fingerprint generation. That is, to establish a common pattern generation method to synthesize the fingerprint information of all samples^33^.

For fingerprint standardization, the fingerprints of 16 batches of *C. yanhusuo* produced in Zhejiang Province were automatically established and matched by using the professional software of ‘Similarity Evaluation System for Chromatographic Fingerprint of Traditional Chinese Medicine’ (v2004 A) (Beijing, China), which was created by the Chinese Pharmacopoeia Committee^34^. First, the reference spectrum was established, the data were cut, the first 5 min and 80 min parts were removed, and multi-point calibration and automatic matching were carried out. In all 16 batches, 13 distinct common peaks were observed between 5 and 80 min, with relatively high intensity and clear separations from the common mode (see Supplementary Fig. S3). Then, the peak areas and retention time of these 13 common peaks were measured (see Supplementary Table S3). The software automatically calculated and generated the simulated ‘average’ chromatogram (as the representative standard chromatogram of fingerprint) by using the median method^34^.

## Conclusions

In view of the inextricable relationships between medicinal plants and their ecological environment in the process of long-term survival competition and natural selection, genetic variation of active components in germplasm resources is an important factor affecting the yield and quality of medicinal plants^35^. Secondary metabolites are the adaptation of plants to the environment in the long-term evolution. To some extent, the formula of “authentic ingredients” has an admirable effect because of the role of “local variety”, i.e., herbs planted in different geographical environments may have different chemical composition content and quality^36^.

In this paper, a HPLC fingerprintand quantitative analysis method was established, which was optimized for the quality evaluation of *C. yanhusuo* from variable origin. The thus obtained variations in chemical fingerprints due to various environmental factors or genotypes were validated by systematic comparison of chromatograms among the 23 samples. Because of the complexity of traditional Chinese medicine components, HPLC is a complex multivariate data set, so the minor differences between very similar chromatograms might therefore be ignored. Hence, chemical pattern recognition methods (SA, HCA, and PCA) were used to reasonably classify *C. yanhusuo*, and to effectively identify it, to explore the impact of geographical location and germplasm resources on the identification of this herb. Owing to the high potential of *C. yanhusuo* rhizomes for food, medicine and value-added product applications, these information are expected to provide help for the identification and quality evaluation of *C. yanhusuo*’s “local variety”.

In the future, we will use modern cultivation techniques of medicinal plants, traditional soil science, ecology, plant nutrition, pharmaceutical analysis and other theories and technologies, combined with ecological environment, germplasm resources, quality of medicinal materials, chemical fingerprint, etc. in the real production areas of Zhejiang Province, we will use the combination of traditional biological morphological characteristics and modern pharmaceutical analysis to screen out high-quality, high-yield, stable ecological germplasm resources of *C. yanhusuo*. Considering the secondary metabolites of *C. yanhusuo*, based on the in-depth deep analysis of the relationship between the physical and chemical properties of soil, the effective nutrient factors and the quality of *C. yanhusuo*, the chemical diversity of this herb was studied according to the environmental factors. The research results will provide a theoretical basis for a complete set of seeding source technology and standardized ecological cultivation technology, which has demonstration and guidance significance for other genuine medicinal materials in Zhejiang Province, and has good social, ecological and economic benefits.

## Methods

### Plant materials

Twenty-three rhizome samples (S1–23) of *C. yanhusuo* were collected from different regions in China in June and classified according to three processing methods, with detailed information provided in Table 1. Among them, S1–8, S22, and S23 were fresh materials collected in Zhejiang, Shanxi, and Jiangsu provinces, respectively. All samples were classified and identified by the corresponding author according to their morphological characteristics, and the certificate specimens were kept in the herbarium of Zhejiang University. S9–20 (boiled materials) and S21 (sulfur-fumigated material) were collected in Zhejiang.

**Table 1:** Details of plant materials collected from different regions in June 2018.

No.	Origin	Processing
S1	Houtang, Dapan, Panan, Zhejiang	Fresh
S2	Kuzhu, Xinwo, Panan, Zhejiang	Fresh
S3	Shangjia, Xinwo, Panan, Zhejiang	Fresh
S4	Kuzhu, Xinwo, Panan, Zhejiang	Fresh
S5	Kuzhu, Xinwo, Panan, Zhejiang	Fresh
S6	Pantan, Lengshui, Panan, Zhejiang	Fresh
S7	Pantan, Lengshui, Panan, Zhejiang	Fresh
S8	Kuzhu, Xinwo, Panan, Zhejiang	Fresh
S9	Kuzhu, Xinwo, Panan, Zhejiang	Boiled
S10	Kuzhu, Xinwo, Panan, Zhejiang	Boiled
S11	Pantan, Lengshui, Panan, Zhejiang	Boiled
S12	Pantan, Lengshui, Panan, Zhejiang	Boiled
S13	Pantan, Lengshui, Panan, Zhejiang	Boiled
S14	Kuzhu, Xinwo, Panan, Zhejiang	Boiled
S15	Liji, Dapan, Panan, Zhejiang	Boiled
S16	Liji, Dapan, Panan, Zhejiang	Boiled
S17	Liji, Dapan, Panan, Zhejiang	Boiled
S18	Liji, Dapan, Panan, Zhejiang	Boiled
S19	Liji, Dapan, Panan, Zhejiang	Boiled
S20	Ganze, Dongyang, Zhejiang	Boiled
S21	Geshan, Dongyang, Zhejiang	Sulfur-fumigated
S22	Hekou, Feng, Baoji, Shanxi	Fresh
S23	Jiaoxie, Haian, Nantong, Jiangsu	Fresh

### Chemical reagents and references

HPLC-grade acetonitrile and methanol were acquired from Fisher Scientific (Pittsburgh, PA, USA). Deionized water is further purified by a Milli-Q Plus water purification system (Millipore Ltd., Bedford, MA, USA) was used throughout. Analytical grade methanol, ethanol, acetic acid, and triethylamine were purchased from Sinopharm Chemical Reagent Co., Ltd. (Shanghai, China). All solutions were filtered through 0.45 μm membranes (Schleicher & Schuell, Dassel, Germany) and degassed by ultrasonication before use.

#### Standard solution preparation

THP (>98%, as determined by the HPLC area normalization method) was obtained from the Shanghai Research and Development Center for Standardization of Traditional Chinese Medicine (Shanghai, China). The reference compound was accurately weighed, dissolved in methanol, and diluted to appropriate concentrations for calibration curve establishment. All stock and working standard solutions were stored at 4 °C before use.

#### Sample solution preparation

Open-air-dried rhizome samples of *C. yanhusuo* were cut into small pieces and ground into powder. Different extraction solvents (methanol and 70 vol% aqueous ethanol with or without dilute acetic acid) and numbers of extractions (1, 2, and 3) were measured for extraction optimization. On the basis of a preliminary test, a 1.0-g finely powdered sample was extracted with 70 vol% aqueous ethanol (2 × 25 mL) upon 30-min ultrasonication. The extract was filtered and evaporated under vacuum. The resulting residue was dissolved in 50 vol% aqueous methanol (10 mL), and the solution was filtered through a 0.45-μm Nylon filter and subjected to HPLC analysis (injection volume = 10 μL).

### Instrumentation and chromatographic conditions

Chromatographic separation was performed on an Agilent 1100 HPLC system (Agilent Technologies, Inc., Santa Clara, CA, USA) equipped with a quaternary pump, an autosampler, a degasser, an automatic thermostatic column compartment, a diode-array detector, and a computer with Chemstation software used for HPLC data analysis. An Agilent HC-C18 reversed-phase column (5 μm, 250 mm × 4.6 mm) together with an Agilent HC-C18 guard column (5 μm, 12.5 mm × 4.6 mm) were used, with the column temperature set to 40 °C. HPLC–diode-array detection was used to assay the purity of reference compounds. The following binary gradient elution system comprising 0.06 vol% triethylamine and 0.6 vol% acetic acid in water (phase A, pH adjusted to 4.0 with formic acid) and acetonitrile (phase B) was used: 0–5 min, 5% B; 5–15 min, 5–10% B; 15–45 min, 10–30% B; 45–65 min, 30–85% B; 65–90 min, 85% B. The above run was followed by a 15-min equilibration period prior to the injection of the next sample. Absorbance was monitored at 280 nm, the mobile phase flow rate equaled 1 mL/min, and on-line UV spectra were recorded in the range of 190–500 nm. The injection volume equaled 10 μL.

### Method validation

The method was validated for repeatability, precision, stability, and accuracy following the International Conference on Harmonization guidelines and several reports on determination analysis^5^. Repeatability was assessed by analyses of five independently prepared extracts of *C. yanhusuo* samples. Precision was validated by injecting the same sample solution five times within a day. The stability test was performed by injecting the identical sample 0, 2, 4, 6, 12, and 24 h after preparation. During this period, the solution was kept at room temperature^37^. For the recovery test, conducted to evaluate accuracy, a 1.0-g powdered sample of *C. yanhusuo* was independently weighed five times and spiked with a known amount of THP (approximately equivalent to 1.0 times the amount contained in the actual plant sample) using the corresponding standard. The spiked samples were extracted and quantified as outlined above.

### Data analysis

For representative chromatographic fingerprint establishment, all samples were analyzed using the presented methods, and the obtained data were exported in AIA format and imported into a professional software package named Computer-Aided Similarity Evaluation System for Chromatographic Fingerprint of Traditional Chinese Medicine (China Committee of Pharmacopeia, 2004A). This system enabled us to accurately determine the similarity of the chemical composition distribution for synchronization and quantitative comparison, as recommended by the State Food and Drug Administration of China^24^. Additionally, PCA and HCA were performed for pre-standardized data using the DPS software package (version 8.01)^38^. Components were supposed to draw the PCA scatter plot. The average-linkage-between-groups method was then applied for HCA, with square Euclidean distance used to establish the distance matrix between observations^39^.

## Supplementary information

Supplementary information.
