# Supplementary material for: Quality Evaluation of Corydalis yanhusuo by High-Performance Liquid Chromatography Fingerprinting Coupled with Multicomponent Quantitative Analysis
Source: Sci Rep. 2020 Mar 19;10:4996. doi: 10.1038/s41598-020-61951-x (PMC7081204; doi:10.1038/s41598-020-61951-x)
Supplement: Supplementary file 1 — Supplementary information. [file 41598_2020_61951_MOESM1_ESM.pdf]

# Quality Evaluation of Corydalis yanhusuo by High -Performance Liquid Chromatography Fingerprinting Coupled with Multicomponent Quantitative Analysis

Yin Lu[1*], Qin Ma[1], Changcun Fu[1], Chuan Chen[2], and Deyong Zhang[1]

[1]College of Biology and Environmental Engineering, Zhejiang Shuren University, Hangzhou, 310015, China.
[2]Hangzhou Botanical Garden, Hangzhou, 310007, China

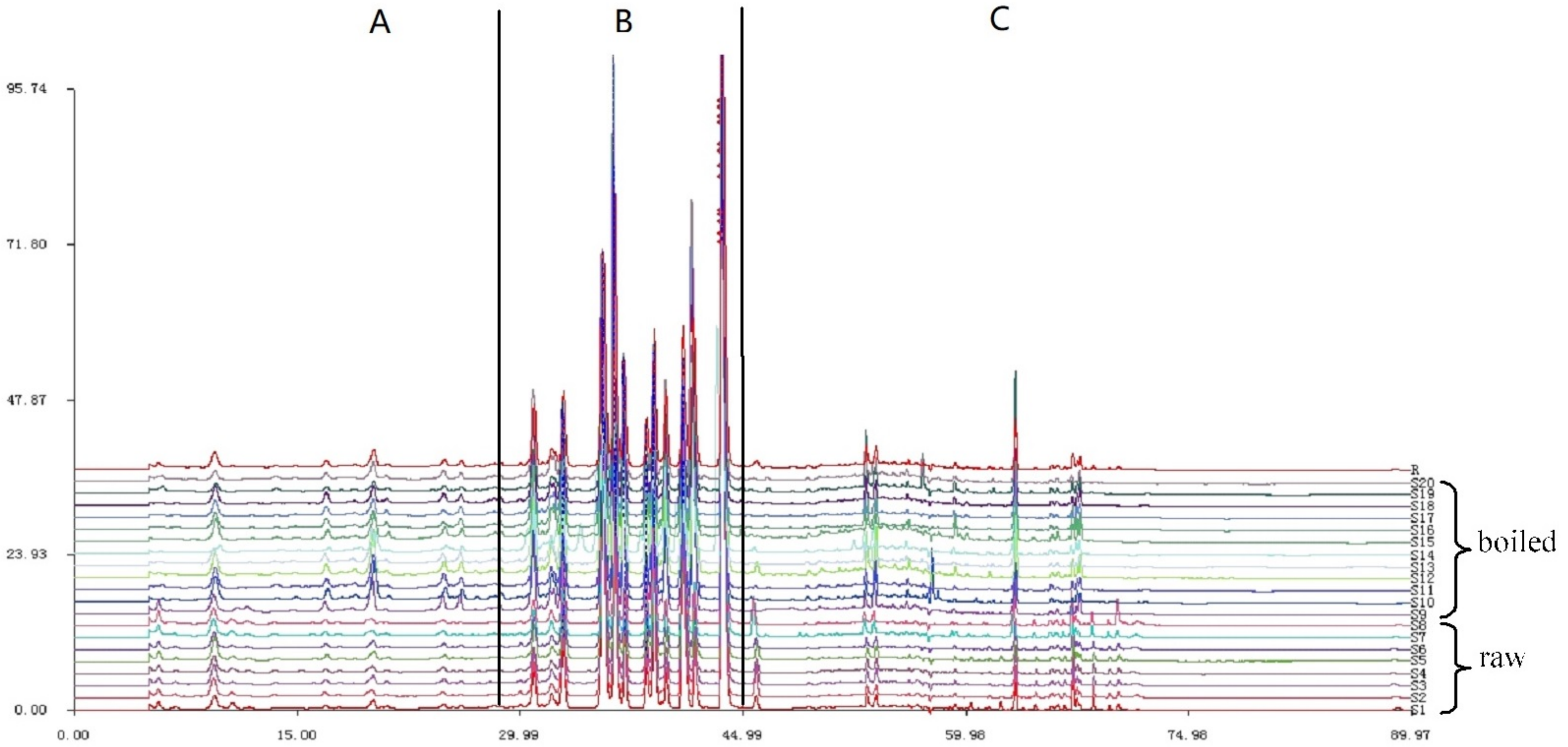


**Supplementary Figure S1.** HPLC fingerprints of fresh and boiled *C. yanhusuo* samples of Zhejiang origin

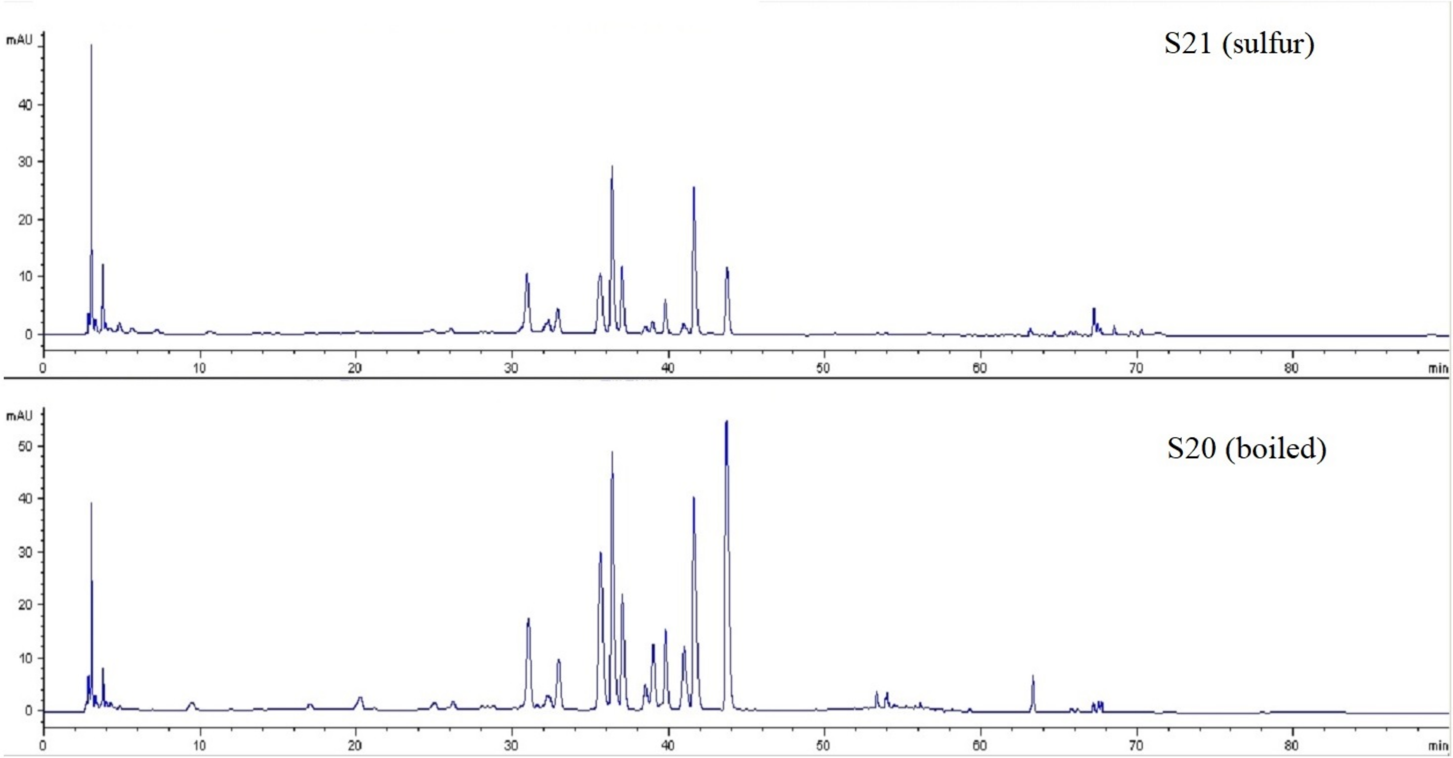


**Supplementary Figure S2.** HPLC fingerprints of *C. yanhusuo* processed by sulfur fumigation and boiling

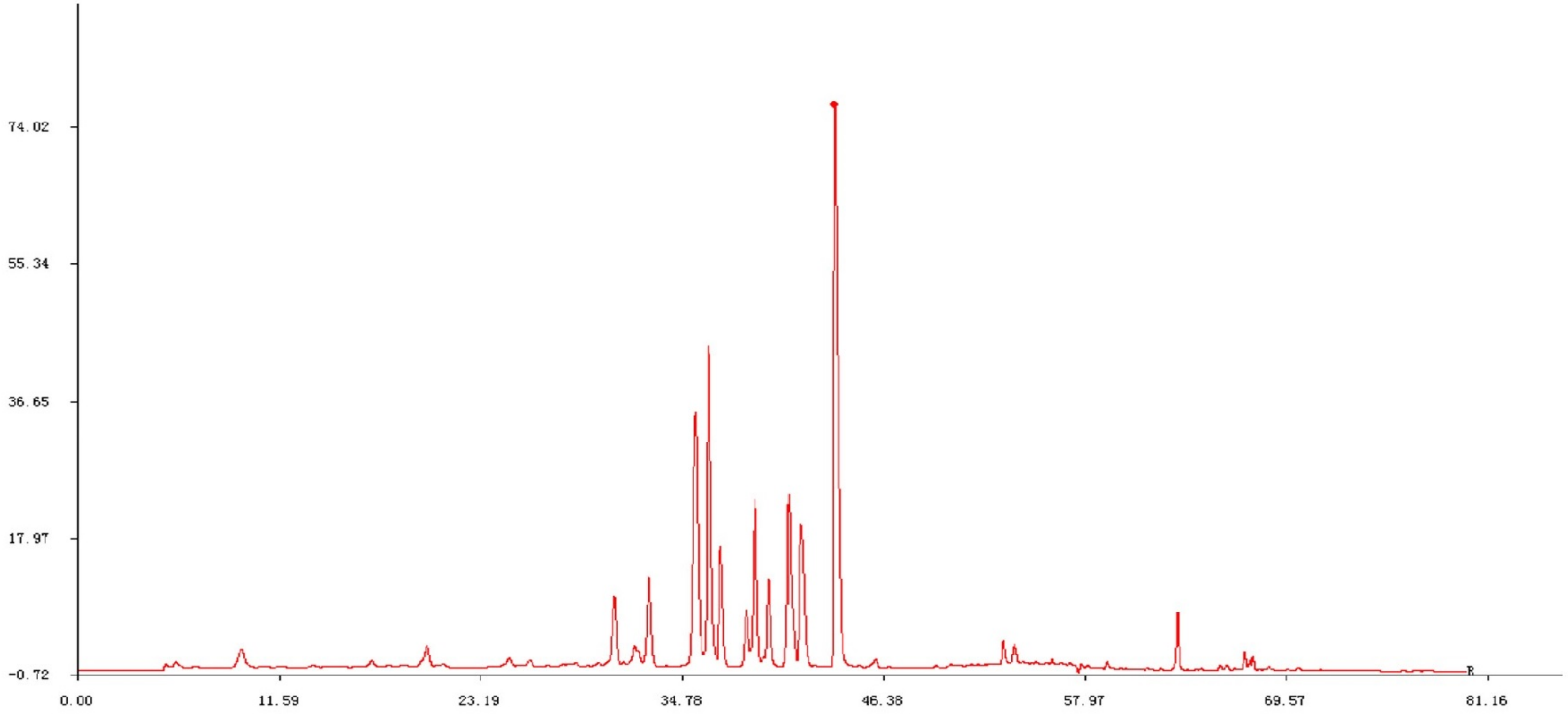


**Supplementary Figure S3.** Common pattern of *C. yanhusuo* fingerprints

**Supplementary Table S1.** Similarity of HPLC fingerprints of *C. yanhusuo*.

| Sample | S1 | S2 | S3 | S4 | S5 | S6 | S7 | S8 | S9 | S10 | S11 | S12 | S13 | S14 | S15 | S16 | S17 | S18 | S19 | S20 | S21 | S22 | S23 | R |
|---|---|---|---|---|---|---|---|---|---|---|---|---|---|---|---|---|---|---|---|---|---|---|---|---|
| S1 | 1 | 0.993 | 0.997 | 0.996 | 0.995 | 0.982 | 0.992 | 0.986 | 0.898 | 0.932 | 0.931 | 0.947 | 0.913 | 0.737 | 0.889 | 0.801 | 0.898 | 0.969 | 0.906 | 0.845 | 0.568 | 0.959 | 0.969 | 0.976 |
| S2 | 0.993 | 1 | 0.996 | 0.996 | 0.996 | 0.975 | 0.994 | 0.992 | 0.873 | 0.904 | 0.909 | 0.923 | 0.886 | 0.71 | 0.861 | 0.772 | 0.88 | 0.951 | 0.88 | 0.814 | 0.522 | 0.936 | 0.945 | 0.96 |
| S3 | 0.997 | 0.996 | 1 | 0.999 | 0.999 | 0.976 | 0.997 | 0.992 | 0.874 | 0.909 | 0.91 | 0.927 | 0.888 | 0.718 | 0.864 | 0.77 | 0.876 | 0.958 | 0.882 | 0.815 | 0.522 | 0.944 | 0.952 | 0.962 |
| S4 | 0.996 | 0.996 | 0.999 | 1 | 0.999 | 0.974 | 0.996 | 0.994 | 0.866 | 0.901 | 0.903 | 0.92 | 0.88 | 0.708 | 0.854 | 0.757 | 0.866 | 0.952 | 0.874 | 0.804 | 0.511 | 0.938 | 0.947 | 0.957 |
| S5 | 0.995 | 0.996 | 0.999 | 0.999 | 1 | 0.976 | 0.996 | 0.994 | 0.87 | 0.907 | 0.908 | 0.923 | 0.883 | 0.712 | 0.857 | 0.761 | 0.871 | 0.956 | 0.877 | 0.809 | 0.512 | 0.94 | 0.948 | 0.961 |
| S6 | 0.982 | 0.975 | 0.976 | 0.974 | 0.976 | 1 | 0.973 | 0.963 | 0.889 | 0.947 | 0.927 | 0.963 | 0.909 | 0.75 | 0.896 | 0.803 | 0.898 | 0.982 | 0.903 | 0.861 | 0.579 | 0.926 | 0.979 | 0.97 |
| S7 | 0.992 | 0.994 | 0.997 | 0.996 | 0.996 | 0.973 | 1 | 0.996 | 0.862 | 0.899 | 0.9 | 0.919 | 0.874 | 0.708 | 0.849 | 0.752 | 0.865 | 0.955 | 0.868 | 0.801 | 0.501 | 0.933 | 0.946 | 0.955 |
| S8 | 0.986 | 0.992 | 0.992 | 0.994 | 0.994 | 0.963 | 0.996 | 1 | 0.835 | 0.874 | 0.877 | 0.894 | 0.848 | 0.676 | 0.816 | 0.712 | 0.835 | 0.937 | 0.839 | 0.764 | 0.455 | 0.915 | 0.926 | 0.939 |
| S9 | 0.898 | 0.873 | 0.874 | 0.866 | 0.87 | 0.889 | 0.862 | 0.835 | 1 | 0.97 | 0.99 | 0.965 | 0.992 | 0.828 | 0.988 | 0.97 | 0.993 | 0.924 | 0.992 | 0.976 | 0.839 | 0.965 | 0.941 | 0.965 |
| S10 | 0.932 | 0.904 | 0.909 | 0.901 | 0.907 | 0.947 | 0.899 | 0.874 | 0.97 | 1 | 0.988 | 0.995 | 0.982 | 0.82 | 0.977 | 0.929 | 0.972 | 0.97 | 0.975 | 0.965 | 0.773 | 0.957 | 0.981 | 0.978 |
| S11 | 0.931 | 0.909 | 0.91 | 0.903 | 0.908 | 0.927 | 0.9 | 0.877 | 0.99 | 0.988 | 1 | 0.984 | 0.992 | 0.82 | 0.984 | 0.947 | 0.989 | 0.956 | 0.987 | 0.967 | 0.788 | 0.973 | 0.965 | 0.983 |
| S12 | 0.947 | 0.923 | 0.927 | 0.92 | 0.923 | 0.963 | 0.919 | 0.894 | 0.965 | 0.995 | 0.984 | 1 | 0.978 | 0.821 | 0.975 | 0.917 | 0.968 | 0.981 | 0.972 | 0.955 | 0.745 | 0.955 | 0.987 | 0.983 |
| S13 | 0.913 | 0.886 | 0.888 | 0.88 | 0.883 | 0.909 | 0.874 | 0.848 | 0.992 | 0.982 | 0.992 | 0.978 | 1 | 0.818 | 0.991 | 0.965 | 0.987 | 0.935 | 0.991 | 0.974 | 0.829 | 0.964 | 0.958 | 0.97 |
| S14 | 0.737 | 0.71 | 0.718 | 0.708 | 0.712 | 0.75 | 0.708 | 0.676 | 0.828 | 0.82 | 0.82 | 0.821 | 0.818 | 1 | 0.833 | 0.803 | 0.825 | 0.797 | 0.828 | 0.828 | 0.694 | 0.791 | 0.797 | 0.802 |
| S15 | 0.889 | 0.861 | 0.864 | 0.854 | 0.857 | 0.896 | 0.849 | 0.816 | 0.988 | 0.977 | 0.984 | 0.975 | 0.991 | 0.833 | 1 | 0.978 | 0.989 | 0.93 | 0.995 | 0.986 | 0.853 | 0.946 | 0.949 | 0.956 |
| S16 | 0.801 | 0.772 | 0.77 | 0.757 | 0.761 | 0.803 | 0.752 | 0.712 | 0.97 | 0.929 | 0.947 | 0.917 | 0.965 | 0.803 | 0.978 | 1 | 0.974 | 0.847 | 0.973 | 0.984 | 0.928 | 0.903 | 0.882 | 0.896 |
| S17 | 0.898 | 0.88 | 0.876 | 0.866 | 0.871 | 0.898 | 0.865 | 0.835 | 0.993 | 0.972 | 0.989 | 0.968 | 0.987 | 0.825 | 0.989 | 0.974 | 1 | 0.931 | 0.991 | 0.982 | 0.834 | 0.957 | 0.944 | 0.965 |
| S18 | 0.969 | 0.951 | 0.958 | 0.952 | 0.956 | 0.982 | 0.955 | 0.937 | 0.924 | 0.97 | 0.956 | 0.981 | 0.935 | 0.797 | 0.93 | 0.847 | 0.931 | 1 | 0.935 | 0.905 | 0.641 | 0.949 | 0.988 | 0.983 |
| S19 | 0.906 | 0.88 | 0.882 | 0.874 | 0.877 | 0.903 | 0.868 | 0.839 | 0.992 | 0.975 | 0.987 | 0.972 | 0.991 | 0.828 | 0.995 | 0.973 | 0.991 | 0.935 | 1 | 0.984 | 0.848 | 0.963 | 0.954 | 0.967 |
| S20 | 0.845 | 0.814 | 0.815 | 0.804 | 0.809 | 0.861 | 0.801 | 0.764 | 0.976 | 0.965 | 0.967 | 0.955 | 0.974 | 0.828 | 0.986 | 0.984 | 0.982 | 0.905 | 0.984 | 1 | 0.898 | 0.924 | 0.929 | 0.932 |
| S21 | 0.568 | 0.522 | 0.522 | 0.511 | 0.512 | 0.579 | 0.501 | 0.455 | 0.839 | 0.773 | 0.788 | 0.745 | 0.829 | 0.694 | 0.853 | 0.928 | 0.834 | 0.641 | 0.848 | 0.898 | 1 | 0.733 | 0.706 | 0.707 |
| S22 | 0.959 | 0.936 | 0.944 | 0.938 | 0.94 | 0.926 | 0.933 | 0.915 | 0.965 | 0.957 | 0.973 | 0.955 | 0.964 | 0.791 | 0.946 | 0.903 | 0.957 | 0.949 | 0.963 | 0.924 | 0.733 | 1 | 0.964 | 0.983 |
| S23 | 0.969 | 0.945 | 0.952 | 0.947 | 0.948 | 0.979 | 0.946 | 0.926 | 0.941 | 0.981 | 0.965 | 0.987 | 0.958 | 0.797 | 0.949 | 0.882 | 0.944 | 0.988 | 0.954 | 0.929 | 0.706 | 0.964 | 1 | 0.987 |
| R | 0.976 | 0.96 | 0.962 | 0.957 | 0.961 | 0.97 | 0.955 | 0.939 | 0.965 | 0.978 | 0.983 | 0.983 | 0.97 | 0.802 | 0.956 | 0.896 | 0.965 | 0.983 | 0.967 | 0.932 | 0.707 | 0.983 | 0.987 | 1 |

**Supplementary Table S2.** Relative retention times of common peaks for all HPLC fingerprints.

| Sample number | Relative retention time of common peaks | | | | | | | | | | | | | | |
|---|---|---|---|---|---|---|---|---|---|---|---|---|---|---|---|
| | Peak1 | Peak2 | Peak3 | Peak4 | Peak5 | Peak6 (s) | Peak7 | Peak8 | Peak9 | Peak10 | Peak11 | Peak12 | Peak13 | Peak14 | Peak15 |
| S1 | 0.835224 | 0.866602 | 0.887854 | 0.961277 | 0.98161 | 1 | 1.039965 | 1.053116 | 1.075448 | 1.104828 | 1.127403 | 1.175794 | 1.708738 | 1.813837 | 1.820291 |
| S2 | 0.834951 | 0.866414 | 0.887633 | 0.961508 | 0.982241 | 1 | 1.039735 | 1.053034 | 1.075199 | 1.104771 | 1.126936 | 1.176186 | 1.710366 | 1.815705 | 1.822057 |
| S3 | 0.834762 | 0.866647 | 0.888228 | 0.961571 | 0.981827 | 1 | 1.039836 | 1.053033 | 1.075209 | 1.104416 | 1.126673 | 1.175433 | 1.710901 | 1.816426 | 1.822674 |
| S4 | 0.834565 | 0.866336 | 0.888014 | 0.961544 | 0.981922 | 1 | 1.039837 | 1.053151 | 1.075289 | 1.10476 | 1.127331 | 1.17599 | 1.712024 | 1.81765 | 1.823875 |
| S5 | 0.835737 | 0.867361 | 0.88922 | 0.962316 | 0.982145 | 1 | 1.040119 | 1.053321 | 1.075504 | 1.104991 | 1.126579 | 1.175409 | 1.711592 | 1.817151 | 1.823373 |
| S6 | 0.835653 | 0.867004 | 0.889057 | 0.96246 | 0.982406 | 1 | 1.039945 | 1.053269 | 1.07516 | 1.105267 | 1.125861 | 1.176266 | 1.709711 | 1.815032 | 1.821275 |
| S7 | 0.834907 | 0.866295 | 0.888082 | 0.961188 | 0.982087 | 1 | 1.039646 | 1.052906 | 1.07472 | 1.104656 | 1.126442 | 1.175744 | 1.702009 | 1.806369 | 1.812932 |
| S9 | 0.83575 | 0.867687 | 0.889069 | 0.961205 | 0.981615 | 1 | 1.041279 | 1.054561 | 1.076699 | 1.108123 | 1.125672 | 1.179909 | 1.709538 | 1.812667 | 1.821387 |
| S10 | 0.837347 | 0.869132 | 0.890348 | 0.962729 | 0.981918 | 1 | 1.041379 | 1.054272 | 1.076137 | 1.107516 | 1.125571 | 1.178194 | 1.711425 | 1.814427 | 1.82313 |
| S11 | 0.836281 | 0.868084 | 0.88988 | 0.962356 | 0.982341 | 1 | 1.04116 | 1.054221 | 1.075558 | 1.107523 | 1.125426 | 1.179052 | 1.712424 | 1.815539 | 1.824247 |
| S12 | 0.83666 | 0.868098 | 0.889518 | 0.962171 | 0.98164 | 1 | 1.04162 | 1.054456 | 1.076309 | 1.107477 | 1.125511 | 1.178261 | 1.714642 | 1.817758 | 1.826505 |
| S13 | 0.83548 | 0.867039 | 0.88861 | 0.961259 | 0.981345 | 1 | 1.04136 | 1.054453 | 1.076564 | 1.08118 | 1.126643 | 1.179774 | 1.70916 | 1.812586 | 1.82109 |
| S15 | 0.837579 | 0.86899 | 0.890211 | 0.960567 | 0.980839 | 1 | 1.041547 | 1.05461 | 1.076508 | 1.107458 | 1.123855 | 1.178844 | 1.716163 | 1.819367 | 1.828012 |
| S17 | 0.835846 | 0.867567 | 0.88903 | 0.961945 | 0.982353 | 1 | 1.041573 | 1.054917 | 1.076217 | 1.107884 | 1.124936 | 1.179554 | 1.713536 | 1.8169 | 1.82548 |
| S18 | 0.835663 | 0.867241 | 0.888859 | 0.962026 | 0.982052 | 1 | 1.039755 | 1.053115 | 1.07449 | 1.105743 | 1.124771 | 1.176401 | 1.708815 | 1.811859 | 1.820523 |
| S19 | 0.835672 | 0.867345 | 0.889036 | 0.961177 | 0.981061 | 1 | 1.040927 | 1.054147 | 1.076431 | 1.107214 | 1.124589 | 1.178978 | 1.708088 | 1.81131 | 1.819727 |
| mean | 0.8358 | 0.8674 | 0.8889 | 0.9617 | 0.9818 | 1 | 1.041 | 1.054 | 1.076 | 1.105 | 1.126 | 1.177 | 1.711 | 1.815 | 1.822 |
| SD | 0.000861 | 0.000873 | 0.000818 | 0.000597 | 0.000462 | 0 | 0.000798 | 0.000717 | 0.000696 | 0.006398 | 0.00103 | 0.001713 | 0.003202 | 0.003203 | 0.003402 |
| SE | 0.000215 | 0.000218 | 0.000205 | 0.000149 | 0.000116 | 0 | 0.0002 | 0.000179 | 0.000174 | 0.0016 | 0.000258 | 0.000428 | 0.000801 | 0.000801 | 0.000851 |

**Supplementary Table S3.** Parameters of the common pattern of *C. yanhusuo*.

| Peak No. | Retention time (min) | Peak height | Peak area |
|---|---|---|---|
| 1 | 30.92955 | 5.300279 | 82.28633 |
| 2 | 32.87864 | 10.82079 | 165.6292 |
| 3 | 35.59822 | 37.6304 | 709.7278 |
| 4 | 36.35099 | 40.23563 | 554.8822 |
| 5 | 37.03188 | 11.43105 | 143.8945 |
| 6 | 38.51232 | 7.698169 | 99.15001 |
| 7 | 38.99852 | 24.28722 | 314.7166 |
| 8 | 39.82639 | 5.226607 | 64.26193 |
| 9 | 40.91402 | 31.88536 | 515.8654 |
| 10 | 41.74979 | 9.914952 | 127.7156 |
| 11 | 43.54177 | 86.48177 | 1501.408 |
| 12 | 63.27769 | 5.440347 | 48.79605 |
| 13 | 67.16992 | 5.779132 | 43.5135 |
